# Suspect identification accuracy from lineups, in the lab and in the field

**DOI:** 10.1186/s41235-025-00670-1

**Published:** 2025-09-19

**Authors:** John T. Wixted, Laura Mickes

**Affiliations:** 1 Department of Psychology, University of California, San Diego, La Jolla, USA; 2 School of Psychological Science, University of Bristol, Bristol, UK

## Abstract

A 2016 field study conducted in collaboration with the Houston Police Department reported that simultaneous lineups were diagnostically superior to sequential lineups, that confidence was strongly predictive of accuracy, and that high-confidence suspect identifications were highly reliable. The study also estimated that most lineups (65%) contained an innocent suspect. Because the innocence or guilt of a suspect in a real police lineup is unknown, however, these conclusions could not be based on direct computations from target-present and target-absent lineups. Instead, they were parameter estimates from a signal detection model fit to the data. A recently published mock-crime laboratory study mirrored key methodological details of the Houston field study, allowing for similar analyses based on direct computations. Here, we compare the results of the two studies and find that they yield similar conclusions. In addition, new model-based analyses of the Houston field data weigh against recent concerns that unfair lineups and other potential biasing factors may have compromised the original model-based estimates. Finally, the lab and field data agree that when encoding conditions are poor (e.g., long viewing distance), witnesses make far fewer high-confidence identifications, but the few witnesses who do express high confidence maintain a high level of accuracy. These findings are consistent with likelihood ratio theories of recognition memory and reinforce a growing consensus that, as encoding conditions become degraded, high-confidence identifications become increasingly rare but are still highly diagnostic. Whether this conclusion holds when conditions are degraded in the extreme is unresolved.

Photo lineups have long been recommended for use in police investigations (Wells et al., 1998, 2020). When conducted in accordance with science-based recommendations, a “pristine” lineup consists of one suspect―someone who the police have independent reason to believe may have committed the crime―and five or more physically similar fillers, blindly administered along with instructions indicating that the perpetrator may or may not be in the lineup. In addition, a witness’s memory of a suspect should be tested only once (Wells et al., 2020; Wixted et al., 2021). To understand identification accuracy when eyewitness memory is initially tested using pristine lineups, researchers have relied on both mock-crime lab studies and real-world field studies.

Each type of study has its advantages and disadvantages. A key advantage of a lab study is that it is known whether the suspect in the lineup is innocent or guilty. This makes it possible to directly compute an important measure known as suspect identification (ID) accuracy, which is the proportion of suspect IDs that land on the guilty suspect. In the simplest case, where half the lineups contain an innocent suspect and half contain a guilty suspect (equal base rates), $\text{Suspect ID Accuracy}=\frac{\text{Number of Guilty Suspect IDs}}{\text{Number of Innocent and Guilty Suspect IDs}}$. This is simply the Bayesian posterior probability of guilt, given that a suspect ID occurred. That is, $\text{Suspect ID Accuracy}=P\left(\text{Guilty Suspect }|\text{ Suspect ID}\right).$ Unlike other lineup accuracy measures sometimes reported (e.g., calibration), this is a formally defensible measure because it directly corresponds to a posterior probability derived from Bayes’ rule. Suspect ID accuracy can be computed separately as a function of confidence―a method known as confidence-accuracy characteristic (CAC) analysis (Mickes, 2015)―to gauge the predictive utility of confidence in a way that maximizes both its theoretical and applied relevance.

However, a widely appreciated disadvantage of most lab studies is that the participants are usually well aware that it is all make-believe. Nevertheless, lab-based studies generally strive to make the participant’s experience as realistic as possible to enhance ecological validity. Using this lab-based approach, a considerable body of research indicates that confidence is predictive of suspect ID accuracy when pristine lineups are used, with high-confidence suspect IDs often exceeding 95% correct (Wixted & Wells, 2017).

The opposite advantages and disadvantages apply to a real-world field study. In a field study, the main advantage is that the identifications are made by actual eyewitnesses tested by the police as part of a real criminal investigation (i.e., it is not make-believe). The main disadvantage is that it is not known whether the suspect in the lineup is innocent or guilty, which makes it impossible to directly compute suspect ID accuracy. To overcome this limitation, Wixted et al. (2016) fit a signal detection model to the Houston Police Department (HPD) field study data (Wells, 2014). Doing so amounted to a theory-based CAC analysis because the best-fitting model provided estimates of suspect ID accuracy separately for each level of confidence. Although these estimates are valid only to the extent that the assumptions of the fitted model are accurate, the confidence-accuracy relationship in the HPD field study (Wixted et al., 2016) was comparable to the confidence-accuracy relationship often found in lab studies (Wixted & Wells, 2017; Wixted et al., 2015).

To evaluate the validity of the theory-based estimates in the HPD field study, one could design a laboratory experiment that mirrors the field study's methodology and then compare the results of the two studies. To the extent that the model-based estimates of suspect ID accuracy from the field study correspond to the directly computed measures of suspect ID accuracy from the similar lab study, it would lend credibility to both approaches. Recently, Ayala et al. (2025) conducted a large-scale lab study (*N* = 6230) that was similar in many ways to the HPD field study. The main purpose of their study was to investigate how the language used by witnesses to justify their identifications could be used by outside observers to distinguish between accurate and inaccurate identification decisions. Here, we analyze their data with a different purpose in mind, namely, to assess the validity of the model-based estimates from the HPD field study.^1^

The methodology used by Ayala et al. (2025) was designed to enhance generalizability to the real world. For example, because both simultaneous and sequential lineups are often used by the police, both presentation formats were used in their study. In the lab, the sequential procedure often terminates the first time a face is identified (Lindsay & Wells, 1985; Steblay et al., 2011), but the police typically present all of the faces, whether or not someone was identified earlier in the sequence. This is why no stopping rule was used in the Ayala et al. lab study. In addition, the lineups were effectively administered in a blind manner (because the study was conducted online), the fillers were description-matched, the suspects and fillers were strangers to the participants, and recommended lineup instructions were used (indicating that the perpetrator may or may not be in the lineup). Ayala et al. also created variability in memory strength across witnesses (an inevitable feature of any field study) by using five simulated viewing distances during encoding (10, 60, 110, 160, or 210 feet). Finally, for each lineup decision, an immediate confidence rating was collected using a 100-point scale.

In short, Ayala et al. (2025) conducted a high-quality large-*N* lab study that happened to share a number of important features with the HPD field study (*N* = 348). For example, similar to the Ayala et al. lab study, the HPD field study also involved the blind administration of both simultaneous and sequential lineups, the sequential lineups also did not use a stopping rule, the suspects and fillers were also strangers to the witnesses, the fillers were also description-matched, and recommended lineup instructions were also used. In addition, the witnesses were naturally exposed to a wide variety of encoding conditions. Finally, for each lineup decision, a confidence rating was immediately collected using a 3-point scale: “weak tentative” (low confidence), “strong tentative” (medium confidence), and “positive” (high confidence).

The HPD field study addressed multiple issues that are of interest to the field of eyewitness identification, including (1) the diagnostic accuracy of simultaneous vs. sequential lineups, (2) the relationship between confidence and accuracy, (3) the base rate of lineups containing a guilty suspect, and (4) the impact of suboptimal estimator variables (Wells, 1978). The latter issue is timely in light of a growing number of articles suggesting that high-confidence suspect ID accuracy might be substantially reduced when the encoding conditions are poor (Davis & Peterson, 2022; Giacona et al., 2021; Lockamyeir et al., 2020; Moore et al., 2024; Sauer et al., 2019). Given their methodological similarities, in what follows, we consider whether suspect ID accuracy measures from the Ayala et al. (2025) lab study―now directly computed based on identifications made from target-absent (TA) and target-present (TP) lineups―correspond to the model-based estimates from the HPD field study.

## Simultaneous vs. sequential lineups

The question of how well eyewitnesses can distinguish between innocent and guilty suspects (i.e., discriminability) in simultaneous versus sequential lineups has been investigated ever since the sequential lineup was first proposed (Lindsay & Wells, 1985). Discriminability is a form of accuracy distinct from the Bayesian posterior probability that a given ID is correct (as in CAC analysis), and it is ideally measured using receiver operating characteristic (ROC) analysis (Wixted & Mickes, 2012).

For many years, non-ROC measures appeared to suggest a sequential superiority effect (see Steblay et al., 2011, for a review). However, the first ROC-based investigation of diagnostic accuracy in these two lineup formats, conducted by Mickes et al. (2012) in a lab study, unexpectedly found evidence of a *simultaneous* advantage. A simultaneous advantage in discriminability is not always observed in lab studies (e.g., Kaesler et al., 2020), but whenever a significant difference has been observed, it has favored the simultaneous procedure (e.g., Dobolyi & Dodson, 2013; see Mickes & Wixted, 2024, for a review). To estimate discriminability in the HPD field study, Wixted et al. (2016) fit a signal detection model to the data shown in Table 1.

**Table 1:** Frequency counts for simultaneous and sequential lineups from the HPD field study (Wixted et al., 2016)

Confidence	Simultaneous lineup			Sequential lineup		
	SID	FID	No ID	SID	FID	No ID
Low	6	24	–	9	30	–
Medium	13	15	–	15	18	–
High	49	9	–	22	8	–
None	–	–	71	–	–	59

SID = suspect identification; FID = filler identification; No ID = no-identification (i.e., lineup rejection). An em dash indicates that the response type was not applicable at that confidence level

**Fig. 1:**
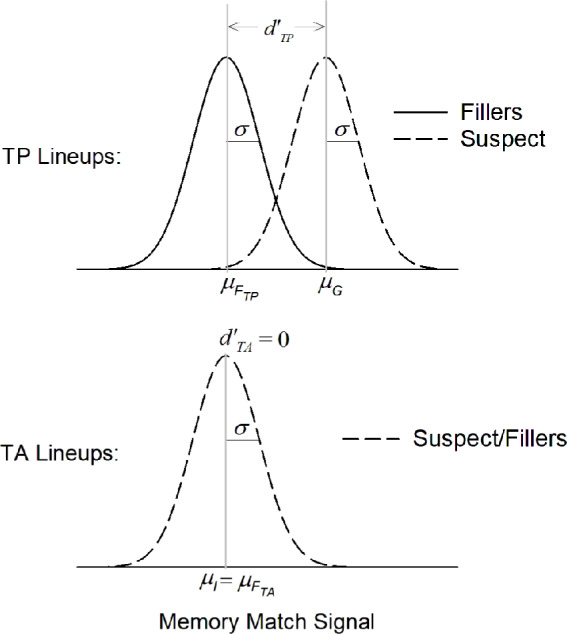
Basic signal detection model for lineups

For this analysis, they used the most straightforward signal detection model for lineups―the Independent Observations model (Fig. 1). In the simplest version of the model, the standard deviation ($\sigma$) for the innocent-suspect/filler distribution is assumed to be equal to that of the guilty suspect distribution. In TP lineups, the mean of the guilty suspect distribution (${\mu }_{G}$) is higher than the mean of the filler distribution (${\mu }_{{F}_{TP}}$). In TA lineups, the mean of the innocent suspect distribution (${\mu }_{I}$) is equal to the mean of the filler distribution (${\mu }_{{F}_{TA}}$), which means that the innocent suspect is, effectively, just another filler (i.e., just another description-matched face that does not match the witness’s memory of the perpetrator). A standard theoretical measure of discriminability is $d{\prime}$. Within lineups, ${d{\prime}}_{TP}=\frac{{\mu }_{G}-{\mu }_{{F}_{TP}}}{\sigma }$ and ${d{\prime}}_{TA}=\frac{{\mu }_{I}-{\mu }_{{F}_{TA}}}{\sigma }$. The mathematical details describing the derivation of the likelihood function for this model (needed to fit the model to empirical data) are described in Wixted et al. (2018).

**Fig. 2:**
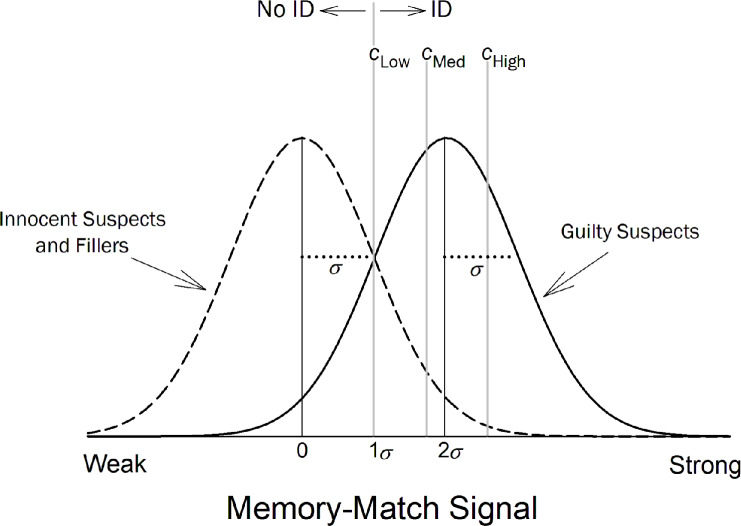
Basic signal detection model for lineups on one axis and with confidence criteria. *Note*. The mean of the guilty suspect distribution is two standard deviations (i.e., two $\sigma$) above the mean of the innocent suspect distribution (i.e., ${\mu }_{G}=2\sigma$ and ${\mu }_{I}=0$). The confidence criterion for making an identification (ID) to the MAX face (${c}_{Low}$) is placed one $\sigma$ above the mean of the innocent-suspect/filler distribution. The confidence criteria for making an ID to the MAX face with low, medium, or high confidence are denoted ${c}_{Low}$, ${c}_{Med}$, and ${c}_{High}$, respectively

The memory signals for fillers in both TP and TA lineups and the innocent suspect in TA lineups are assumed to be drawn from the same distribution, so this model can be more simply represented as two distributions (innocent suspects and fillers vs. guilty suspects), with confidence criteria now added to illustrate the different levels of confidence made for positive IDs (Fig. 2). The mean of the distribution for innocent suspects and fillers (i.e., for ${\mu }_{I}$, ${\mu }_{{F}_{TA}}$, and ${\mu }_{{F}_{TP}}$ in Fig. 1) is set to 0, and the mean of the guilty suspect distribution (i.e., ${\mu }_{G}$ in Fig. 1) is placed two $\sigma$ higher than that (i.e., ${\mu }_{G}=2\sigma$). Thus, in the model depicted here, the ability to distinguish innocent from guilty suspects is ${d}{\prime}=\frac{{\mu }_{G}-{\mu }_{I}}{\sigma }=2$.

The model assumes that the witness’s decision is based on the face in the lineup that generates the strongest (i.e., MAX) memory match signal. If the strength of the MAX face exceeds the decision criterion (the ${c}_{Low}$ criterion in Fig. 2), that face will be identified with some degree of confidence. Confidence in the ID is determined by the highest confidence criterion the memory-match signal exceeds (e.g., if its strength exceeds the ${c}_{Med}$ criterion but not the ${c}_{High}$ criterion, the face is identified with medium confidence). If the strength of even the MAX face does not exceed the lowest decision criterion (${c}_{Low}$), the lineup is rejected. We used this model to fit data from both simultaneous and sequential lineups. This model makes sense even for sequential lineups because, in both the HPD field study and the Ayala et al. (2025) lab study, a no stopping rule was used. Thus, witnesses could always base their decision on the MAX face, even if they had identified a less familiar face earlier in the lineup. When a stopping rule is used, however, a different and more complex model is needed (see, for example, Kaesler et al., 2020; Kellen & McAdoo, 2022; Wilson et al., 2019).

This model fit the HPD field data shown in Table 1 well, ${\chi }^{2}(6)=3.29$, and the maximum-likelihood parameter estimates are shown in Table 2. A base-rate parameter ($\pi$) was needed for this because field data are aggregated over TA and TP lineups. Thus, the model needs to estimate the proportion of each lineup type. This estimate came to 35% (i.e., $\pi =0.35$), which means that an estimated 65% of the suspects in the HPD lineups were innocent. Constraining $\pi$ to 0.50 (i.e., equal base rates) significantly worsened the fit, ${\chi }^{2}\left(1\right)=6.90, p<.01$. The fact that the model converges on a unique estimate of this parameter is remarkable and, to say the least, counterintuitive.^2^ We consider this base rate estimate in more detail later.

**Table 2:** Maximum-likelihood parameter estimates obtained from fitting the model depicted in Figs. 1 and 2 to the HPD field data

Format	*µ*_*G*_	*c*_*L*ow_	*c*_Med_	*c*_High_	π
SIM	2.93	1.27	1.73	2.28	0.35
SEQ	2.03	1.27	1.73	2.28	0.35

The criterion (*c*) and base-rate (π) parameters (but not the ${\mu }_{G}$ parameter) were constrained to be equal across lineup formats; their identical values are repeated for clarity

In the model we fit to the data, the mean of the guilty suspect distribution, ${\mu }_{G}$ (Fig. 1), is equal to discriminability, $d{\prime}$, because ${d}{\prime}=\frac{{\mu }_{G}-{\mu }_{I}}{\sigma }$. Thus, with ${\mu }_{I}$ and $\sigma$ defined to equal 0 and 1 respectively, ${d}{\prime}={\mu }_{G}$. As shown in Table 2, ${d}{\prime}$ in the HPD field data was estimated to be significantly higher for simultaneous lineups (${d}{\prime}=2.93$) compared to sequential lineups (${d}{\prime}=2.03$), ${\chi }^{2}\left(1\right)=5.01, p= 0.025$. Theoretically, a larger $d{\prime}$ means that the memory signals generated by guilty suspects across TP lineups overlap to a lesser degree with the memory signals generated by innocent suspects across TA lineups.

With regard to the confidence criteria, the estimates for ${c}_{Low},{c}_{Med},$ and ${c}_{High}$ were nearly identical for the simultaneous and sequential formats, so they were constrained to be equal across lineup formats without significantly worsening the fit. An important implication of this confidence-criterion equivalence is that the estimated false alarm rates (i.e., the rate of innocent suspect IDs from TA lineups) did not differ across lineup conditions. This outcome contrasts with a longstanding idea that sequential lineups reduce the false alarm rate compared to simultaneous lineups (e.g., Steblay et al., 2011).

We next fit this model to the Ayala et al. (2025) lab results, with the data pooled over the five viewing distance conditions, estimating the following parameters using maximum likelihood estimation: ${\mu }_{G},{c}_{Low},{c}_{Med},$ and ${c}_{High}$ (4 parameters per lineup format). No base-rate parameter was needed this time because the lab data are not aggregated over TP and TA lineups. The data from their study are shown in Table 3, separately for TP and TA lineups.

**Table 3:** Frequency counts for simultaneous and sequential lineups from Ayala et al. (2025)

Confidence	Simultaneous lineup						Sequential lineup					
	TP			TA			TP			TA		
	SID	FID	No ID	SID	FID	No ID	SID	FID	No ID	SID	FID	No ID
Low	262	151	–	65	323	–	328	225	–	54	268	–
Medium	219	43	–	20	100	–	337	109	–	26	132	–
High	414	17	–	9	43	–	519	63	–	14	69	–
None	–	–	284	–	–	823	–	–	539	–	–	774

SID = suspect identification; FID = filler identification; No ID = no-identification response. Low = confidence ratings of 0–60, Medium = confidence ratings of 70–80, and High = confidence ratings of 90–100. Confidence ratings for No IDs were available, but we collapsed over them because no such ratings were available in the HPD field study. There was no designated innocent suspect in target-absent lineups, so 1/6 of target-absent filler IDs were treated as innocent suspect IDs and 5/6 as filler IDs (the same approach was used to perform the CAC analyses reported here). An em dash indicates no observations

The model fit these data reasonably well, ${\chi }_{SIM}^{2}(5)=17.5$ and ${\chi }_{SEQ}^{2}(5)=11.1$, and the maximum-likelihood parameter estimates shown in Table 4 reflect the same discriminability pattern observed in the HPD field study (here again, ${{d}{\prime}=\mu }_{G}$). That is, as noted by Ayala et al. (2025), discriminability for simultaneous lineups (${d}{\prime}=1.96$) significantly exceeded that for sequential lineups (${d}{\prime}=1.69$), ${\chi }^{2}\left(1\right)=31.4, p< 0.001$. Although the estimates for ${c}_{Low},{c}_{Med},$ and ${c}_{High}$ were similar across lineup formats, constraining them to be equal significantly worsened the fit. Interestingly, the criterion estimates were slightly lower (i.e., slightly more liberal) for the sequential procedure, corresponding to the slightly *higher* false alarm rates compared to the simultaneous procedure. That said, the overall false alarm rate was nearly identical for the two procedures.

**Table 4:** Maximum likelihood signal-detection-based estimates for simultaneous and sequential lineups from fitting the Ayala et al. (2025) data (pooled over viewing distance conditions)

Format	*µ*_*G*_	*c*_*L*ow_	*c*_Med_	*c*_High_
SIM	1.96	1.41	2.06	2.51
SEQ	1.69	1.39	1.90	2.37

All parameter estimates are in units of σ (i.e., standard deviation units)

**Fig. 3:**
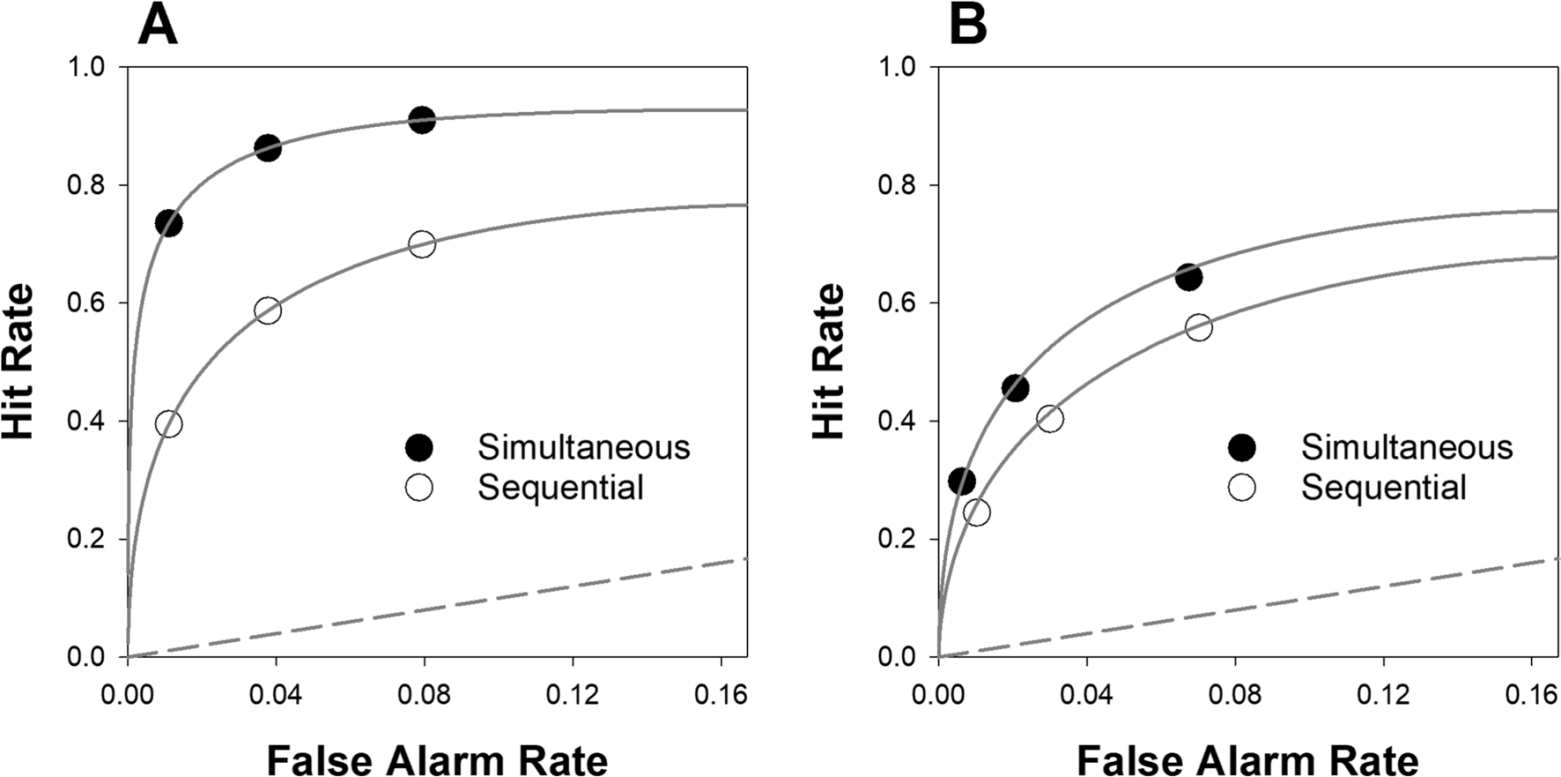
Receiver operating characteristic (ROC) data and fits for the simultaneous and sequential conditions of the HPD field study (**A**) and Ayala et al., 2025 (**B**). *Note*. Both the ROC points and the curves in panel A were generated by the best-fitting signal detection model to the HPD data. In panel B, the ROC points were directly computed from the Ayala et al. data, estimating the false alarm rates by dividing TA filler IDs by lineup size.

Figure 3A shows the ROC data implied by the model fit to the HPD field data (i.e., both the data points and the smooth curves were generated by a signal detection model, using the parameter estimates shown in Table 2), and Fig. 3B shows the ROC data that were directly computed from the Ayala et al. (2025) lab study (with the smooth curves generated by a signal detection model using the parameter estimates shown in Table 4).

3 The smooth curves in panel B were generated by the best-fitting signal detection model. The dashed line in both panels represents chance performance. The Hit Rate is the proportion of suspect IDs in TP lineups, and the False Alarm rate is the estimated proportion of suspect IDs in TA lineups

Both ROCs visually illustrate the higher discriminability achieved through the use of simultaneous lineups. Note also that the ROC points are more-or-less vertically arranged across lineup formats in both cases, which means that the false alarm rates were similar for simultaneous and sequential lineups. In short, the HPD field study and Ayala et al. lab study converge on the conclusion that $d{\prime}$ is enhanced using a simultaneous compared to a sequential lineup.

The ROC data exhibit higher discriminability for the HPD field study compared to the Ayala et al. (2025) lab data (i.e., the HPD field study ROC curves are farther above the diagonal line of chance performance), and this is true for both simultaneous and sequential lineups. Conceivably, this difference is explained by the fact that, in the Ayala et al. lab study, a similar number of participants experienced very poor viewing conditions with a low $d{\prime}$ (e.g., 210 feet) and very good viewing conditions with a high $d{\prime}$ (e.g., 10 feet). By contrast, the police may preferentially administer lineups to witnesses who claim to have had a good look at the perpetrator (i.e., witnesses who had more favorable viewing conditions). In the Ayala et al. study, the $d{\prime}$ values for the more favorable viewing conditions (10 feet and 60 feet) were comparable to the $d{\prime}$ values (and to the correspondingly high ROC curves) estimated from the HPD field study data.

## Confidence vs. accuracy (CAC analysis)

**Fig. 4:**
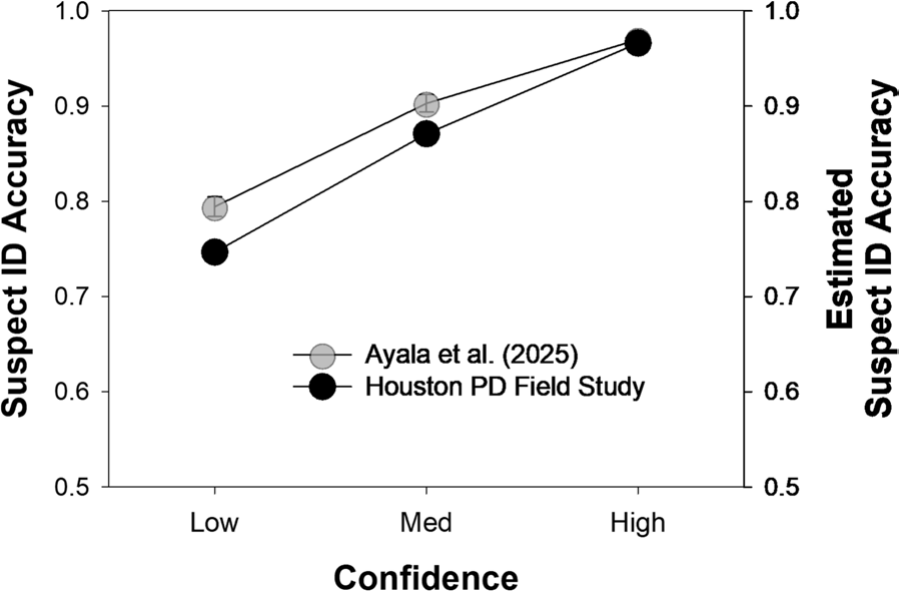
Suspect ID accuracy for the Ayala et al. (2025) lab study and estimated suspect ID accuracy for the Houston PD field study. *Note*. Suspect ID accuracy for the Ayala et al. (2025) lab study (gray symbols, left y-axis) and the Houston PD field study (black symbols, right y-axis). Both y-axes share the same scale (0.5–1.0), but the labels differ because the values were computed differently. Also, the Ayala et al. data were slightly adjusted to correspond to a 50% base rate of TP lineups, and the model-based Houston PD estimates depict estimated accuracy under the same base rate (50%)

Next, we combined the simultaneous and sequential lineup data from the Ayala et al. (2025) lab study and directly computed suspect ID accuracy as a function of confidence (low, medium, and high). Once again, innocent suspect IDs were estimated by dividing TA filler IDs by lineup size. We then compared the results to the model-based estimates for the HPD field study data (also combined across simultaneous and sequential lineups) reported by Wixted et al. (2016). As noted earlier, Wixted et al. estimated suspect ID accuracy by fitting the Independent Observations model illustrated in Figs. 1 and 2 to the data. Figure 4 shows the results of both studies, and it is apparent that they are quite similar. Moreover, in both cases, confidence is predictive of accuracy, and high-confidence accuracy is approximately 97% correct.

The observed convergence shown in Fig. 4 lends credibility to the results of both studies. Moreover, the results indicate that, among the witnesses tested in these studies, an uncontaminated memory, when first tested using a pristine lineup, yielded reliable information.^4^

## Base rates

Lab studies typically use 50% TA and 50% TP lineups, but the base rate of TA vs. TP lineups in real-world police investigations is unknown. Yet, as noted earlier, when the model illustrated in Figs. 1 and 2 is fit to collapsed real-world data, it can provide an estimate of the base rate of guilty suspects ($\pi$), which turned out to be 0.35 for the HPD field data (i.e., 35% of the lineups contained a guilty suspect and 65% contained an innocent suspect).

So far as we know, this remains the first and only quantitative estimate of the base rate of TP lineups in any real-world jurisdiction. To investigate its validity, we applied the same analysis to the Ayala et al. (2025) results after collapsing their data over the TA and TP lineups to make them comparable to field data. In that study, the simultaneous lineups were evenly divided between TA lineups (*N* = 1383) and TP lineups (*N* = 1390). In other words, the base rate of guilt was approximately 0.50. The sequential lineups differed in that there were fewer TA lineups (*N* = 1337) than TP lineups (*N* = 2120). In other words, the base rate of guilt for the sequential lineups was 0.61. The extra TP lineups were used to ensure that the guilty suspect appeared equally often in the six sequential lineup positions. The uncollapsed data from this study were shown earlier in Table 3. Table 5 shows the same data except now they are collapsed over TA and TP lineups, as if the data came from a field study.

**Table 5:** Frequency counts for simultaneous and sequential lineups from Ayala et al. (2025) collapsed over TA and TP lineups

Confidence	Simultaneous			Sequential		
	SID	FID	No ID	SID	FID	No ID
Low	327	474	–	382	493	–
Medium	239	143	–	363	241	–
High	423	60	–	533	132	–
None	–	–	1107	–	–	1313

Low = confidence ratings of 0–60, Medium = confidence ratings of 70–80, and High = confidence ratings of 90–100

The top two rows of Table 6 show the parameter estimates obtained by fitting the Independent Observations model to the collapsed data shown in Table 5. Note that the ${\mu }_{G}$ (i.e., $d{\prime}$) estimates remain largely the same as those obtained from fitting the uncollapsed data (shown earlier in Table 4) and continue to favor the simultaneous procedure. In addition, the estimate of π for simultaneous lineups (0.45) is reasonably close to the known value of 0.50, and the estimate for the sequential lineups (0.60) is almost equal to the known value of 0.61.

**Table 6:** Maximum likelihood signal-detection-based estimates for simultaneous and sequential lineups from fitting the Ayala et al. (2025) data collapsed across target-present and target-absent lineups in two ways (base rate of guilt = 0.50 or 0.35)

Format	Base rate	*µ*_*G*_	*c*_*L*ow_	*c*_Med_	*c*_High_	π
SIM	0.50	2.15	1.40	2.09	2.57	0.45
SEQ	0.50	1.72	1.39	1.91	2.39	0.60
SIM	0.35	2.10	1.40	2.06	2.55	0.32
SEQ	0.35	1.64	1.37	1.88	2.35	0.37

The bottom two rows of Table 6 show the parameter estimates from fitting the same model to the Ayala et al. (2025) data after collapsing over TA and TP lineups but now in such a way that only 35% of the data came from TP lineups and 65% from TA lineups. For this analysis, the known base rate in the collapsed Ayala et al. data corresponds to the estimated base rate in the HPD field data (for which $\pi =0.35$). When the model was fit to these data, the ${\mu }_{G}$ and confidence criteria estimates remain similar to the estimates obtained after analyzing both the uncollapsed data (Table 4) and collapsed data (top two rows of Table 6), and the base rate estimates are close to the known value of 0.35. The successful recovery of known base rates from the collapsed Ayala et al. data supports the validity of the 0.35 base rate estimate derived from the necessarily collapsed HPD field data. These results also fit with an earlier investigation of this issue reported by Cohen et al. (2020) using other datasets.

**Fig. 5:**
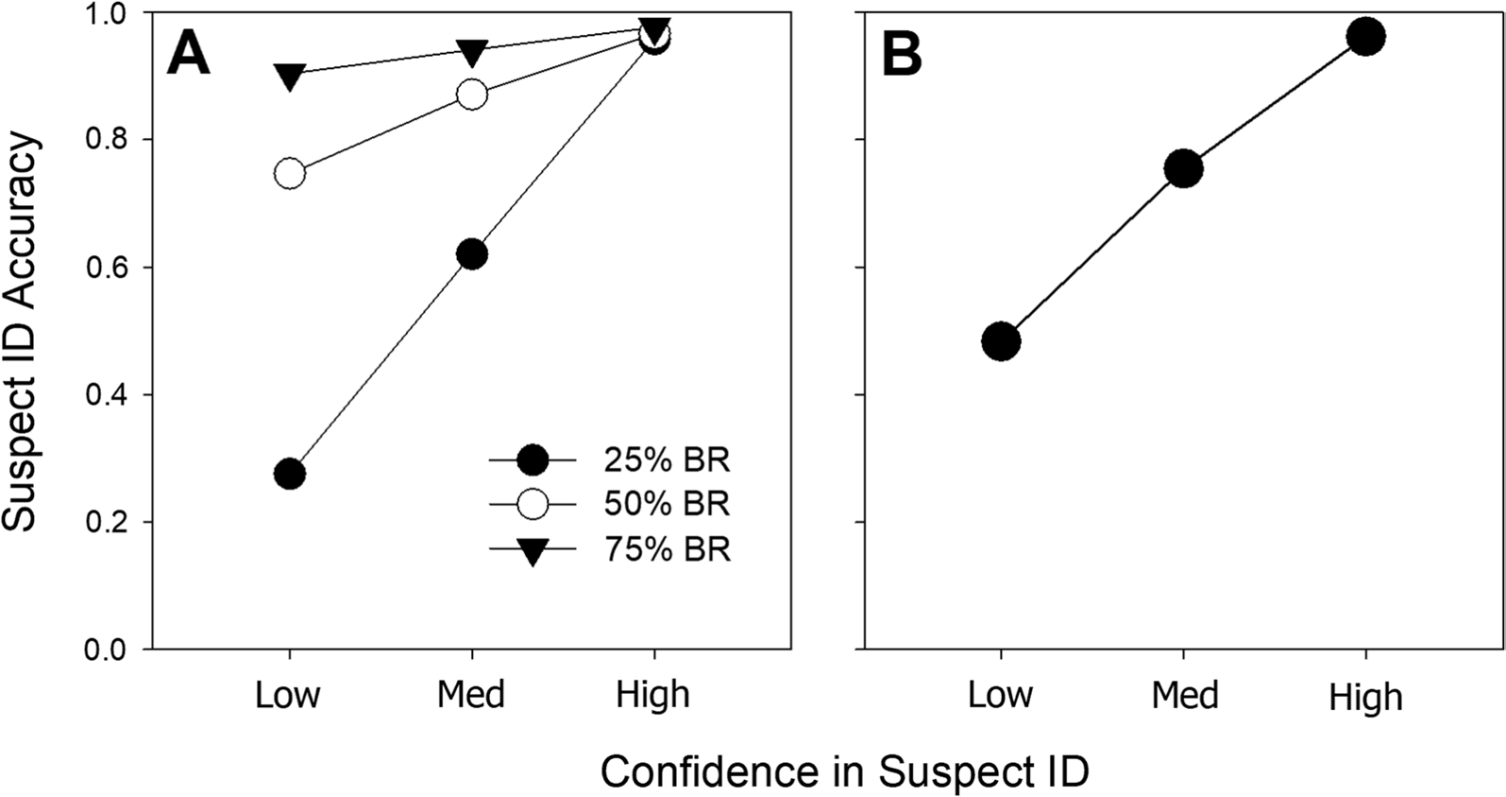
Estimates of suspect ID accuracy from the HPD field study (collapsed over simultaneous and sequential lineups) for different base rates of guilt (**A**) and for the estimated base rate of 35% (**B**). *Note*. Suspect ID accuracy is measured as the proportion of suspect IDs that are correct. BR = base rate of guilt

Estimating base rates from field studies is important because it is a determinant of suspect ID accuracy—that is, the Bayesian posterior probability that a positive ID is correct. According to Bayes’ theorem, lower base rates of guilty suspects yield lower suspect ID accuracy. For example, Wixted et al. (2016) presented model-based estimates of suspect ID accuracy in HPD field study for base rates of guilt ranging from 25 to 75%, and we reproduce those results here in Fig. 5. Low-confidence accuracy is more affected by variations in the base rate than high-confidence accuracy, though both are affected. The 35% base rate estimate in the HPD field study is the only science-based estimate that we know of for any jurisdiction, and it may provide a useful starting point for the prosecution and defense to further debate real-world cases.

## Suspect bias

The model illustrated earlier in Figs. 1 and 2 assume fair lineups (i.e., no suspect bias), in which case innocent suspects in TA lineups are functionally equivalent to fillers. This is a safe assumption about the Ayala et al. (2025) lineups, but Steblay and Wells (2020) and Fitzgerald et al. (2023) expressed concerns that the HPD lineups may have been biased against innocent suspects. Their concern was that the innocent suspects in TA lineups may have often been the best match to the witness’s description of the perpetrator. However, as reported by Wixted et al. (2016), 48 mock witnesses provided with the witness’s description of the perpetrator were only able to identify the suspect at chance accuracy. A different set of HPD lineups that were found to be somewhat biased using this method were considered only in the supplementary material precisely because they were not found to be perfectly fair.

Then again, even if HPD lineups were fair in the sense that everyone in the lineup matched the witness’s description of the perpetrator, various other factors could still have biased the HPD lineups against the innocent suspect (Fitzgerald et al., 2023; Smalarz, 2021). For example, the witness’s memory may have already been contaminated by finding the innocent suspect's photo on social media, or the suspect may have been selected based on resemblance to a publicized photo, etc. Cohen et al. (2020) found that parameter estimates derived from fitting the model illustrated in Fig. 1 to collapsed field data are valid only under unbiased lineup conditions. If the HPD lineups were actually biased against innocent suspects for one reason or another, contrary to the model's assumptions, the resulting parameter estimates would be correspondingly inaccurate.

**Fig. 6:**
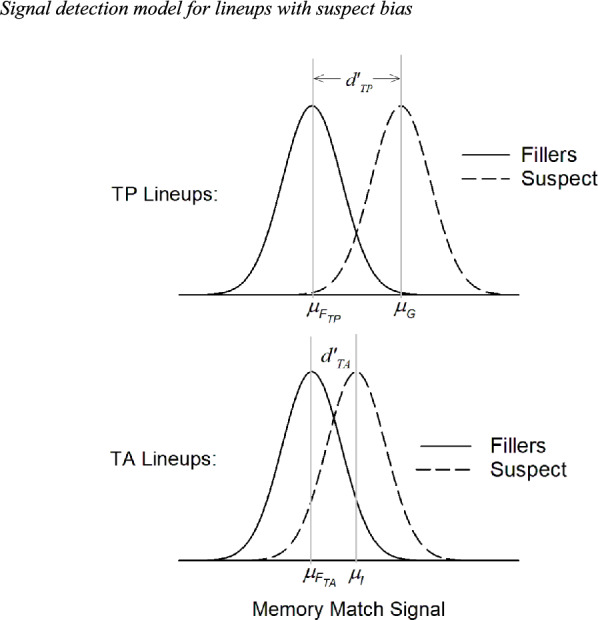
Signal detection model for lineups with suspect bias. *Note*. As in Fig. 1, in TP lineups, the mean of the guilty suspect distribution (${\mu }_{G}$) is higher than the mean of the filler distribution (${\mu }_{{F}_{TP}}$). In TA lineups, the mean of the innocent suspect distribution (${\mu }_{I}$) is now also higher than the mean of the filler distribution (${\mu }_{{F}_{TA}}$), reflective of suspect biasing factors. As before, ${d{\prime}}_{TP}=\frac{{\mu }_{G}-{\mu }_{{F}_{TP}}}{\sigma }$ and ${d{\prime}}_{TA}=\frac{{\mu }_{I}-{\mu }_{{F}_{TA}}}{\sigma }$

A signal detection model that reflects the presence of a suspect biasing factor in TA lineups is illustrated in Fig. 6. Unlike the model that was previously used to evaluate the HPD field data (Fig. 1), now, the memory signal for an innocent suspect in a TA lineup is drawn from a distribution with a higher mean (${\mu }_{I}$) than the distribution of memory signals for the TA fillers (${\mu }_{{F}_{TA}}$). Whereas we previously fit the model shown in Fig. 1 to the HPD field data (Wixted et al., 2016), here, we fit the model depicted in Fig. 6 to the data to directly evaluate the possibility that the lineups were biased against innocent suspects.

To ensure that this model could recover the correct parameter values when fit to unfair lineup data collapsed over TA and TP lineups (an issue that has not been previously investigated), we first generated simulated data from an Independent Observations signal detection model corresponding to Fig. 6 with ${\mu }_{G}=2.5$, ${\mu }_{I}=1.0{, \mu }_{{F}_{TP}}={\mu }_{{F}_{TA}}=0$, and $\sigma =1$. Thus, for these simulated data, ${d{\prime}}_{TP}=2.50$ and ${d{\prime}}_{TA}=1.00$ (instead of ${d{\prime}}_{TA}=0$, the value for unbiased lineups). We also set the confidence criteria to ${c}_{Low}$ = 0.67, ${c}_{Med}$ = 1.67, and ${c}_{High}$ = 2.77. With these programmed parameter values in place, we simulated 100,000 decisions from TP lineups and 100,000 decisions from TA lineups. These simulated decisions consisted of suspect IDs made with low, medium, or high confidence, filler IDs made with low, medium, or high confidence, and lineup rejections, separately for TA and TP lineups.

We then collapsed the data over the TA and TP lineups (as would be true in a field study). The simulated data were first collapsed evenly across TP and TA lineups by using all 100,000 simulated decisions from TP lineups and all 100,000 simulated decisions from TA lineups combined (base rate = 0.50). We then collapsed the data unevenly by using the first 35,000 simulated decisions from TP lineups and the first 65,000 simulated decisions from TA lineups combined (base rate = 0.35). Finally, the model shown in Fig. 6 was fit to both sets of collapsed data, and the results are shown in Table 7. It is clear that the model can accurately recover the parameters from even collapsed data involving biased lineups.

**Table 7:** Programmed (Actual) parameter values and maximum likelihood parameter estimates (Estimated) from fitting biased simulated data collapsed across TP and TA lineups

Parameters	*µ*_*G*_	*µ*_*I*_	*c*_*L*ow_	*c*_Med_	*c*_High_	π
Actual	2.50	1.00	0.67	1.67	2.77	0.50
Estimated	2.57	1.06	0.67	1.67	2.77	0.46
Actual	2.50	1.00	0.67	1.67	2.77	0.35
Estimated	2.60	1.05	0.67	1.67	2.78	0.31

The top two rows show the fits to the collapsed data with a base rate of 0.50 (i.e., half the lineups were TP lineups with a guilty suspect) and the bottom two rows show the fits to the collapsed data with a base rate of 0.35 (i.e., 35% of the lineups were TP lineups with a guilty suspect, and 65% were TA lineups with an innocent suspect)

We are now in a position to answer the question of interest: Is there evidence of suspect bias in the HPD field study? To find out, we fit the model in Fig. 6 to the HPD field data in the same way as Wixted et al. (2016) did except that, this time, ${\mu }_{I}$ was allowed to differ from ${\mu }_{{F}_{TA}}$ (as before, ${\mu }_{{F}_{TA}}$ and ${\mu }_{{F}_{TP}}$ were both set to 0). If the lineups were biased, the estimate of ${\mu }_{I}$ should come out to be greater than 0, as was true of the fits to the known-to-be-biased simulated data in Table 7. However, the results instead replicated the original estimates exactly, including the same base rate estimate of 0.35. In other words, although free to vary, the fit was not significantly or even slightly affected by allowing ${\mu }_{I}$ to be greater than ${\mu }_{{F}_{TA}}$. If anything, a slight non-significant improvement was obtained with ${\mu }_{I}$ estimated to be less than 0 (the opposite of suspect bias).

We also tried fixing ${\mu }_{I}$ to a small value greater than 0, such as 0.25 (consistent with a small amount of suspect bias) while allowing the other parameters to vary, but the fit was always worse than that obtained with ${\mu }_{I}$ fixed at 0 (or allowed to take on values less than 0). We interpret these results as weighing against speculation about the possible existence of suspect bias in the HPD study. Instead, the remarkably high accuracy rate of confident suspect IDs (approaching 97% correct) withstands the scrutiny of both this new model-based analysis and the comparative analysis with the directly computed high-confidence accuracy values in the Ayala et al. (2025) data.^5^

## Estimator variables and likelihood ratio theory

The analyses reported to this point support the idea that in the lab and in the real world, when memory is tested using recommended procedures for a lineup, high-confidence suspect IDs are highly reliable. Standing somewhat in opposition to this perspective is recent work suggesting that high-confidence suspect IDs are unreliable under certain circumstances, even when a witness’s uncontaminated memory is first tested using a pristine lineup. According to this alternative perspective, when the encoding conditions at the time of the crime (i.e., estimator variables) are sufficiently poor, then high confidence is no longer indicative of high accuracy.

If suboptimal estimator variables substantially reduce the accuracy of high-confidence suspect IDs even when pristine lineups are used, the high-accuracy associated with high-confidence suspect IDs in the HPD field study would be somewhat puzzling because, in that study, multiple estimator variables were suboptimal. For example, all of the crimes were robberies, and a weapon was present in most cases. It therefore stands to reason that weapon focus (Loftus et al., 1987) was often an issue and that stress was often high. In addition, most of the IDs were cross race. Few would consider these to be optimal estimator variable conditions. Why, then, did estimated high-confidence accuracy turn out to be so high?

*Likelihood Ratio Theory*. Semmler et al. (2018) proposed a likelihood ratio theory of eyewitness identification according to which witnesses appreciate the negative effects of suboptimal estimator variables and adjust their confidence ratings accordingly. Thus, for example, as $d{\prime}$ decreases (e.g., due to poor lighting, long distance, weapon focus, etc.), witnesses appreciate the suboptimal nature of the encoding conditions and demand that a face generate a stronger memory-match signal before making a confident identification (thereby maintaining high accuracy). In other words, they shift their criterion for making a high-confidence ID to a more conservative setting. The data reported by Ayala et al. (2025) can be used to evaluate that theory.

**Fig. 7:**
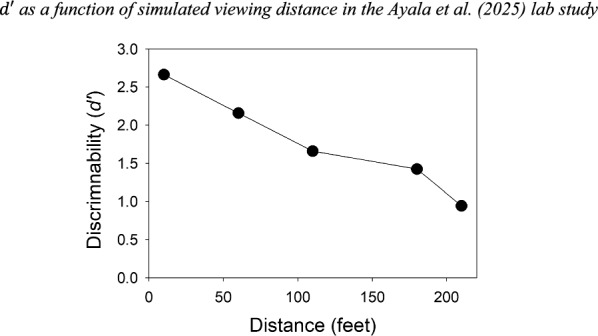
$d^{\prime}$ as a function of simulated viewing distance in the Ayala et al. (2025) lab study

As noted earlier, Ayala et al. (2025) used 5 different viewing-distance conditions. According to the values reported in their paper, and as would be expected, *d'* was high when distance was 10 feet (we might consider this to be an optimal estimator variable condition) and dropped to much lower levels as distance increased to 210 feet (i.e., as the viewing-distance estimator variable became increasingly suboptimal). The ${d}{\prime}$ results are shown in Fig. 7.

**Fig. 8:**
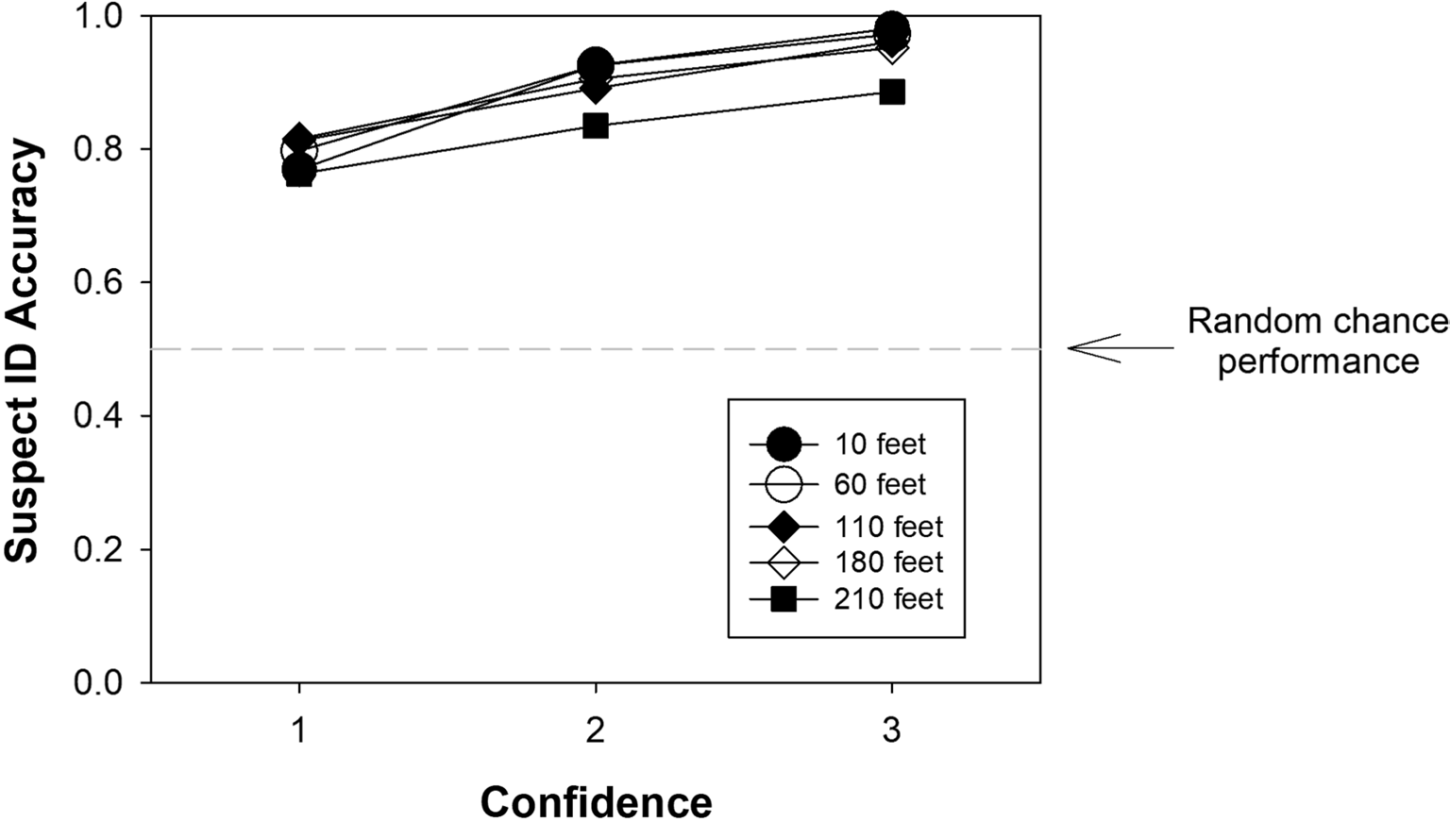
Suspect ID accuracy as a function of confidence for the Ayala et al. (2025) lab study computed separately for each viewing distance condition. *Note*. 1 = low confidence, 2 = medium confidence, and 3 = high confidence. Because base rates affect suspect ID accuracy, the data have been slightly adjusted to correspond to a base rate of 50% (i.e., half TP lineups and half TA lineups).

How did the dramatically decreasing discriminability as a function of viewing distance affect the confidence-accuracy relationship? As shown in Fig. 8, and as predicted by likelihood ratio theory, not very much:

6In all five viewing-distance conditions, suspect ID accuracy was well above chance, even when the witnesses expressed low confidence. Moreover, high-confidence accuracy remained extremely high across the various distance conditions and only showed signs of dropping off in the worst estimator variable condition (210 feet). Even then, high-confidence accuracy was still 89% correct, far above the random-chance value of 50% correct. On the surface, this last result offers at least some support to recent claims that suspect ID accuracy decreases appreciably when the estimator variables become suboptimal in the extreme. If so, the results would deviate to some degree from the strictest version of likelihood ratio theory, according to which eyewitnesses are perfect likelihood ratio computers such that high-confidence accuracy remains unchanged as $d{\prime}$ approaches 0. However, there is an important issue to keep in mind, one that we consider next.

**Fig. 9:**
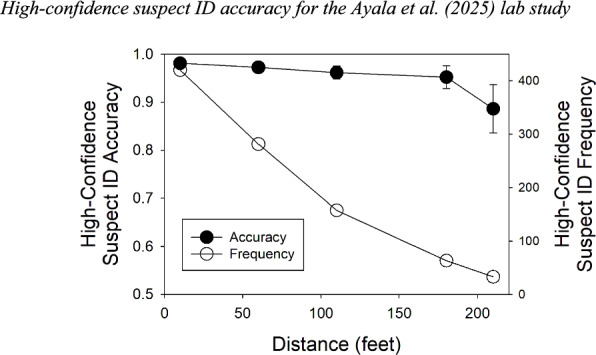
High-confidence suspect ID accuracy for the Ayala et al. (2025) lab study. *Note*. High-confidence suspect ID accuracy corresponds to the left y-axis; the number of high-confidence suspect IDs to the right y-axis. As before, the accuracy scores have been slightly adjusted to correspond to a 50% base rate scenario (i.e., 50% TP lineups and 50% TA lineups)

Figure 9 sheds light on how accuracy generally remained so high despite the increasingly suboptimal estimator variable conditions. Plotted on the left axis is high-confidence suspect ID accuracy (filled symbols) for each of the 5 viewing-distance conditions. Plotted on the right axis is the total number of high-confidence suspect IDs (number correct + estimated number incorrect) for those same 5 conditions (open symbols). Although high-confidence accuracy remains high across the board, with some drop off at 210 feet, the number of high-confidence suspect IDs drops dramatically, from over $n=400$ at a distance of 10 feet to only $n=33$ at 210 feet. It is the two phenomena considered together―that is, high accuracy across the board while the number of high-confidence IDs declines precipitously with worsening estimator variable conditions―that reveal how sensitive eyewitnesses are to worsening encoding conditions. Indeed, in the 210 feet condition, less than 3% of participants made a high-confidence ID, whereas 33% did in the 10 feet condition.

Similar considerations apply to Experiment 2 of Lockamyeir et al. (2020), which was much like that of Ayala et al. (2025) except that only simultaneous lineups were used. The experiment involved a large number of participants (*N* = 3762) randomly assigned to one of three simulated viewing distances (3 m, 10 m, and 20 m, or ~ 10 feet, ~ 33 feet, and ~ 66 feet). We estimated the relevant data from their tables and figures using WebPlotDigitizer and again estimated the number of innocent suspect IDs by dividing TA filler IDs by the lineup size of 6 (because there was no designated innocent suspect).

The identification task in Lockamyeir et al. (2020) was apparently difficult because even at the shortest distance of 3 m $d{\prime}$ was only 1.58. The number of high-confidence suspect IDs (i.e., number guilty + estimated number innocent) in the 3 m condition was approximately $n=121$, which amounts to only 9% of the tested participants. Even so, high-confidence accuracy was ~ 97% correct. However, in the 20 m condition (long viewing distance), performance dropped to near-chance levels (${d}{\prime}=0.29$). Now, only $n=19$ high-confidence suspect IDs were made―that is, only 1.6% of the 1194 participants tested confidently identified a suspect―and accuracy was only 63% correct.

While Lockamyeir et al. (2020) emphasized this low accuracy rate, it is important to remember that the more robust finding is that witnesses rarely make high-confidence suspect identifications under poor viewing conditions. This pattern is directly predicted by likelihood ratio theory and may help explain why, in real-world DNA exoneration cases involving high-confidence misidentifications at trial (Garrett, 2011), it is hard to find cases where the witness made the same high-confidence error on the initial test (Mickes et al., 2025). In other words, high-confidence suspect IDs under poor estimator variable conditions theoretically should be—and may in fact be—rare.

**Fig. 10:**
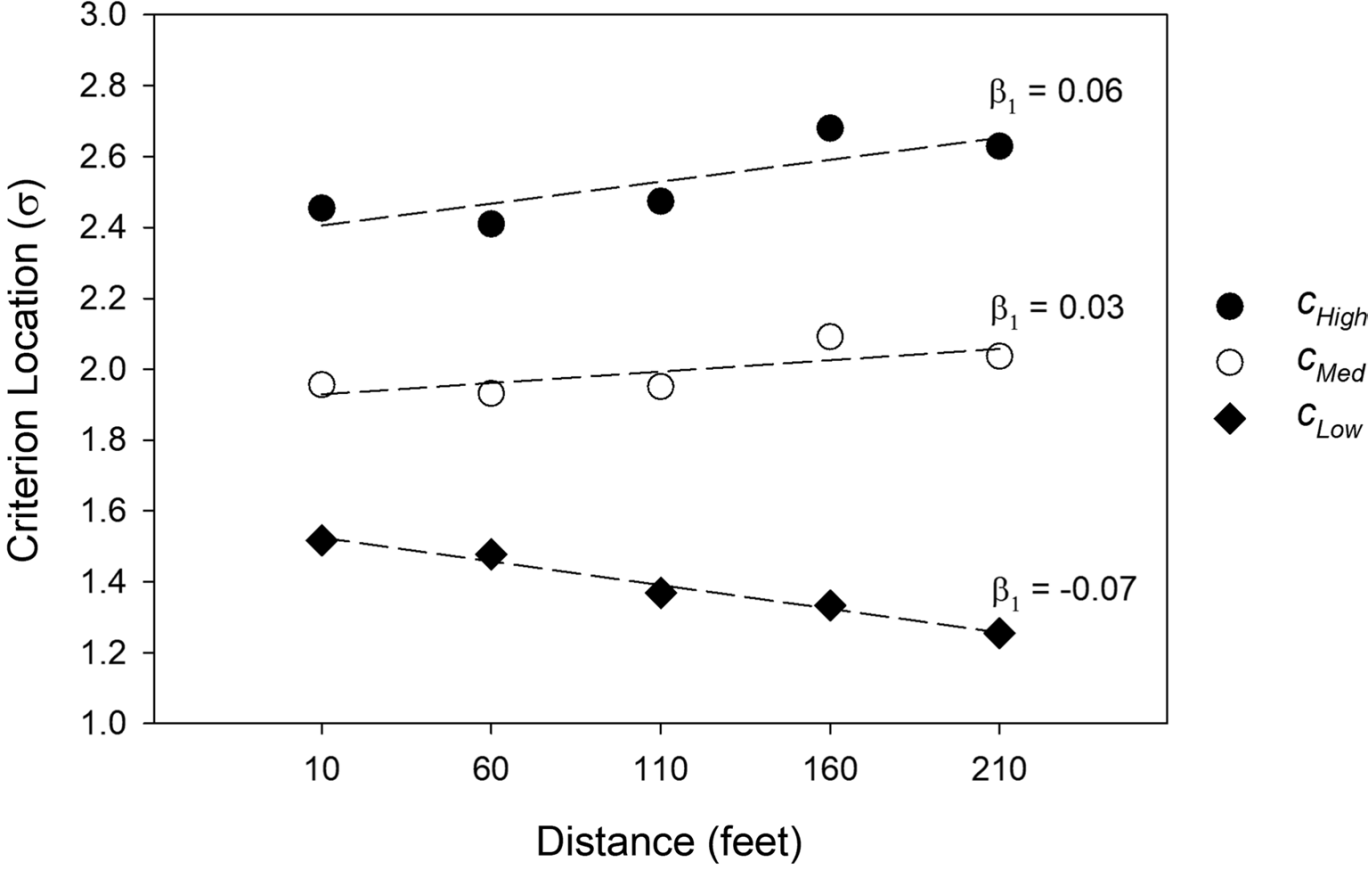
Maximum likelihood estimates of the confidence criteria (${\text{c}}_{\text{Low}}$, ${\text{c}}_{\text{Med}}$, and ${\text{c}}_{\text{High}}$) as a function of viewing distance in the Ayala et al. (2025) study

To illustrate this issue in a way that makes its connection to likelihood ratio theory more apparent, Fig. 10 plots the location of the estimated confidence criteria obtained by fitting the Independent Observations model separately to each distance condition in the Ayala et al. (2025) dataset. The pattern was similar for simultaneous and sequential lineups, so the estimates are averaged across lineup format.

As distance increased (and $d{\prime}$ decreased), the overall decision criterion (${c}_{Low}$) shifted to a lower position (closer to 0). This shift to the left is commonly observed in recognition memory studies, including in list-learning paradigms, and it is a standard explanation for the “mirror effect” (Glanzer & Adams, 1985). The mirror effect refers to the common empirical observation that as $d{\prime}$ decreases, the hit rate decreases, and because of the leftward criterion shift, the false alarm rate simultaneously increases. However, and this is the critical point, the other confidence criteria shift to the right (i.e., to more conservative settings) as $d{\prime}$ decreases, which means that a stronger memory signal is required to identify a face with high confidence when the encoding conditions become worse. The slopes of the regression lines (${\beta }_{1}$ values) are shown for each confidence criterion to underscore the fact that the criteria fan out as $d{\prime}$ decreases, which is the same pattern reported by Semmler et al. (2018). The fanning pattern evident in Fig. 10 is a signature prediction of the likelihood ratio theory of recognition memory decision-making (Stretch & Wixted, 1998). The basic idea is that participants adjust their confidence criteria in such a way as to maintain essentially the same level of accuracy despite variations in $d{\prime}$.^7^

**Fig. 11:**
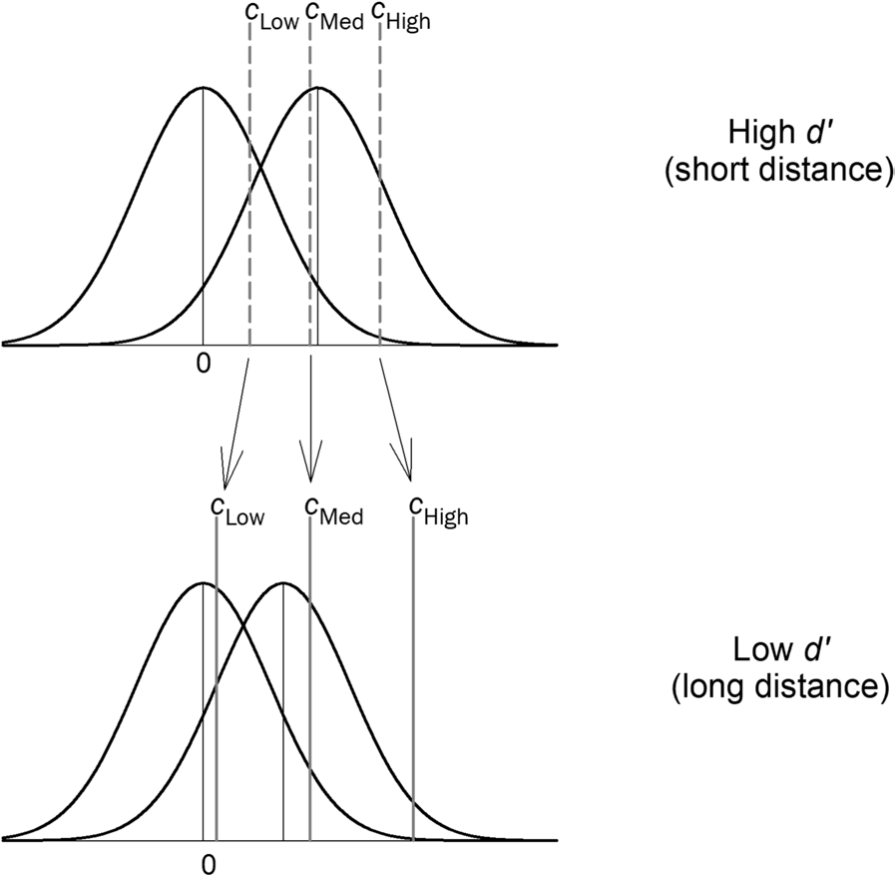
An illustration of how the confidence criteria fan out as a $d^{\prime}$ decreases, as predicted by a constant likelihood ratio model

Figure 11 shows a signal detection model for two different estimator variable conditions (high $d{\prime}$ and low $d{\prime}$), and it illustrates how the confidence criteria shift according to the parameter estimates shown in Fig. 10. Notice that the overall decision criterion (${c}_{Low}$) shifts to the left as $d{\prime}$ decreases (top to bottom), whereas the criterion for declaring high confidence (${c}_{High}$) shifts to the right. The rightward shift of ${c}_{High}$ maintains the accuracy of ever-fewer high-confidence suspect IDs as the estimator variables worsen and $d{\prime}$ decreases accordingly.

These considerations add context to the high-confidence suspect ID accuracy score of 63% reported by Lockamyeir et al. (2020). When $d{\prime}$ decreases and witnesses become more conservative about making a high-confidence ID, very few such IDs occur. Under such conditions, only a few noncompliant or otherwise problematic mock-crime participants would exert a disproportionately large effect on the accuracy of high-confidence suspect IDs. The lab study results certainly warrant further investigation of this matter in a real-world field study, but, at least in our view, they do not yet warrant any strong conclusions about the reliability of confident suspect IDs made by actual eyewitnesses to a crime. Indeed, as described next, the available real-world data from the HPD field study support a different conclusion.

*Estimator Variables in the HPD Field Study*. To investigate the role of estimator variables on eyewitness accuracy in the real world, Giacona et al. (2021) reanalyzed the same HPD field study data that we have been considering here. Because suspect ID accuracy could not be directly computed, they instead analyzed the proportion of all identifications that were suspect IDs―that is, suspect IDs / (suspect IDs + filler IDs)―computed separately for different levels of confidence. An advantage of this statistic is that it can be directly computed from field data, without the aid of a signal detection model. However, its relevance is questionable.

Giacona et al. (2021) used this measure to investigate Semmler et al.’s (2018) claim that suboptimal viewing conditions do not appreciably decrease the accuracy of high-confidence suspect identifications. The HPD field study documented the status of 7 estimator variables for each case (e.g., exposure duration, clarity of view, lighting, etc.), and Giacona et al. (2021) divided each case into those associated with a “good view” (0 or 1 suboptimal estimator variable) vs. those with a “poor view” (2 or more suboptimal estimator variables). For identifications made with high confidence, p(SID|ID) was approximately 0.87 for the cases with a good view and declined to 0.71 for those with a poor view. These findings were taken to support their overall message, which was summarized in the article’s public significance statement as follows:

**Previous research has suggested that estimator variables (e.g., lighting, viewing conditions) might be less important when the eyewitness is highly confident. However, the current study illustrates that poor viewing conditions, relative to good viewing conditions, reduce the accuracy of eyewitnesses, even with high-confidence witnesses and under conditions that are otherwise pristine. This finding is particularly important when eyewitness confidence is used by police and courts to weight [sic] witness accuracy (p. 256).**

However, p(SID|ID) is not a measure that usefully informs the court about what it cares the most about, namely suspect ID accuracy (i.e., the probability that a confidently identified suspect is guilty). Indeed, it is intuitively misleading. Using an estimate based on theory, Wixted and Mickes (2022) pointed out that p(SID|ID) findings like those reported by Giacona et al. (2021) are consistent with suspect ID accuracy values that exceed 95% correct in both estimator variable conditions. If so, it would provide important context to the claim that poor viewing conditions reduce the accuracy of confident suspect IDs from pristine lineups. We now address the same issue empirically, using the Ayala et al. (2025) lab data as a guide.

**Fig. 12:**
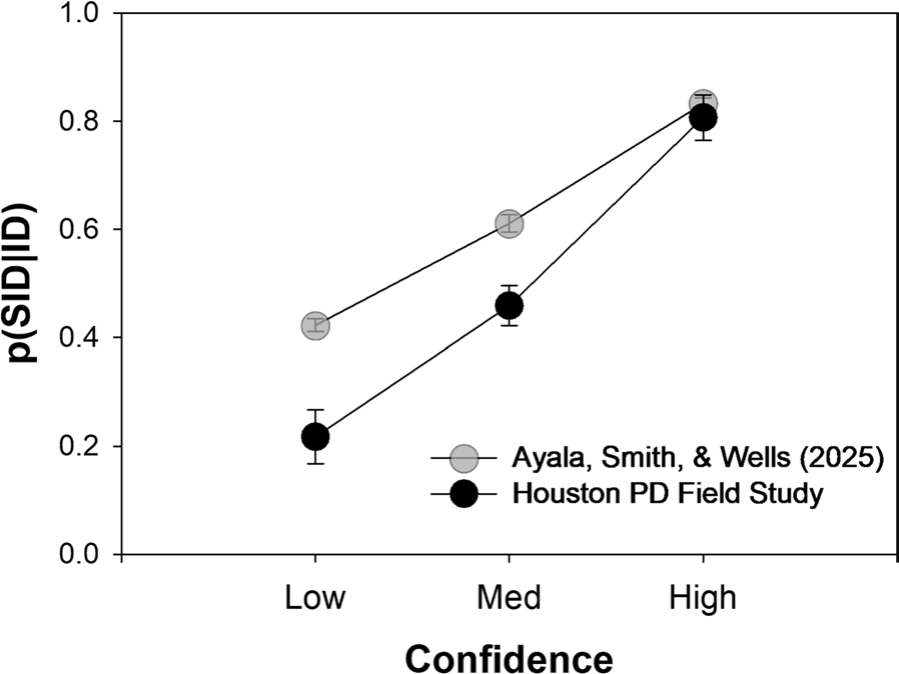
Probability that a positive ID lands on a suspect ID, p(SID|ID), in Ayala et al. (2025) and the HPD field study

Figure 12 shows the p(SID|ID) measure for both the HPD field study and the Ayala et al. (2025) lab data after collapsing over TA and TP lineups (to again make the lab data like field study data). The proportion of high-confidence IDs that landed on the suspect as opposed to a filler was similar in both studies (approximately 80%), but the data from the two studies diverged somewhat as confidence decreased. Still, the overall pattern is similar across the lab and field studies.

**Fig. 13:**
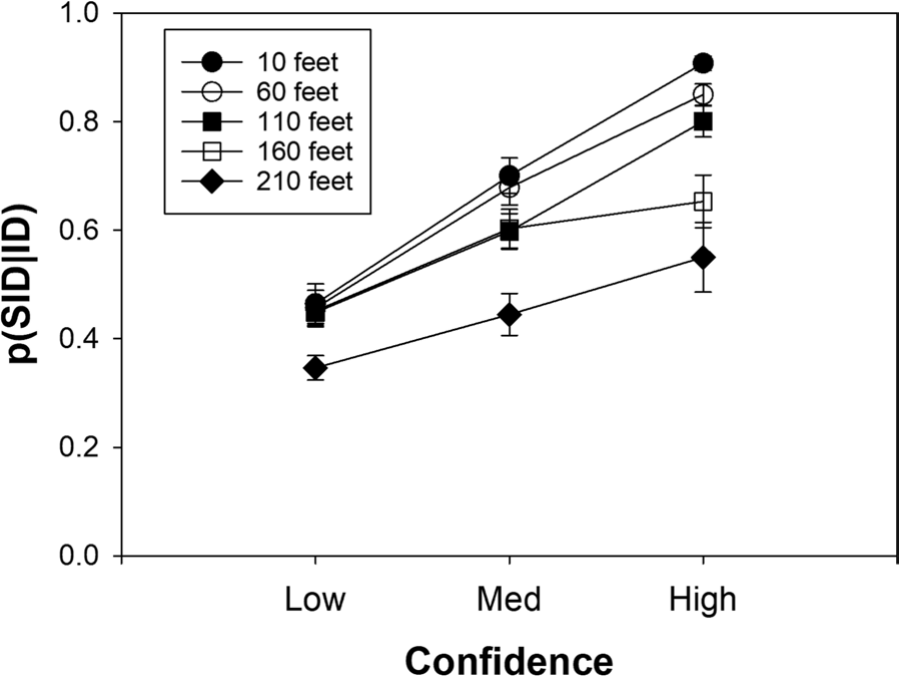
Probability that a positive ID lands on a suspect ID, p(SID|ID), as a function of viewing distance in Ayala et al. (2025)

In the Ayala et al. (2025) lab data, we can break this statistic down by the five viewing-distance conditions, as shown in Fig. 13. Clearly, and somewhat alarmingly at first glance, for identifications made with high confidence (rightmost points), p(SID|ID) decreases dramatically as viewing distance increases. Indeed, at the longest viewing distance of 210 feet, almost half of the high-confidence identifications are known errors (i.e., they are made to fillers). On the surface, the high proportion of confident IDs that are known errors in the suboptimal estimator variable conditions might seem like an indictment of the reliability of confident eyewitness identifications under those conditions. However, appearances can be misleading.

Combining data from the 10 feet and 60 feet conditions of Ayala et al. (2025) yields a high-confidence p(SID|ID) value of 0.88, similar to the corresponding estimate from the “good view” cases from the HPD field study (0.87). In addition, combining data from 110 and 160 feet conditions yields a high-confidence p(SID|ID) value of 0.73, similar to the corresponding estimate from the “poor view” cases from the HPD field study (0.71). Instead of relying on theory, using the Ayala et al. data, we can directly compute suspect ID accuracy (the actual measure of interest) for those two viewing conditions.

Critically, the high-confidence suspect ID accuracy scores for the “good view” and “poor view” conditions are 98% correct and 96% correct, respectively. This small difference might be real, but it is negligible in magnitude and is not even close to being statistically significant (*z* = 1.08, *p* = 0.274). Thus, using the Ayala et al. (2025) empirical data as an interpretive guide yields the same conclusion reached by Wixted and Mickes (2022) using a theory-based analysis: high-confidence identification accuracy in the HPD field study remains high whether the encoding conditions are optimal, where p(SID|ID) = 0.88, or suboptimal, where p(SID|ID) = 0.73.

Stretch and Wixted (1998) argued that although the general pattern of criterion shifts as $d{\prime}$ changes conformed to the predictions of likelihood ratio theory (as is true of the pattern shown in Fig. 10 here), humans are not *perfect* likelihood ratio computers. Thus, as $d{\prime}$ decreases, high-confidence accuracy would be expected to also decline somewhat, and it does (Fig. 9). However, it seems important not to overstate that effect, at least not until real-world data indicate otherwise. In addition, from a scientific perspective, it seems important to recognize the dramatic reduction in the probability of a high-confidence suspect ID as estimator variable conditions worsen. This pattern might help to explain why high-confidence misidentifications on the initial test have rarely been documented in real-world DNA exoneration cases.

## Conclusion

Here, we considered suspect ID accuracy when eyewitness memory is tested for the first time (before contamination) using a pristine simultaneous or sequential lineup. Overall, direct computations from the Ayala et al. lab study and model-based estimates from the HPD field study largely agree: simultaneous lineups yield higher discriminability than sequential lineups, confidence predicts accuracy, high-confidence accuracy is not substantially affected by poor estimator variable conditions, consistent with likelihood ratio theory, and, critically, a signal detection model can be used to estimate the base rate of guilt in actual police lineups.

Another lab study (Nyman et al., 2019), similar to Ayala et al. (2025), also varied viewing distance and tested participants with either simultaneous or sequential lineups. They found that confidence strongly predicted suspect ID accuracy, that high-confidence IDs were highly accurate across viewing distances of 5–40 m (~ 16 to ~ 130 feet), and that $d{\prime}$ for simultaneous lineups exceeded that for sequential lineups. This study used a stopping rule for sequential lineups and used 8-person rather than 6-person lineups, design features that differ from the HPD field study, which is why we focused here on the Ayala et al. lab data. Nevertheless, the results reported by Nyman et al. (2019) largely converge with and further reinforce our main conclusions.

When CAC analysis was first applied to the pristine-lineup literature (Mickes, 2015; Wixted & Wells, 2017; Wixted et al., 2015), findings long thought to illustrate the error-prone nature of high-confidence IDs were instead found to reflect accuracy rates often exceeding 95% correct. Today, it is widely acknowledged that confidence is more informative than previously believed when a pristine lineup is used (e.g., Seale-Carlisle et al., 2024). Still, some recent studies suggest that confident IDs are error-prone under suboptimal estimator conditions, even with a pristine lineup. If so, experts might reasonably argue at trial that confident IDs are generally unreliable, since in most cases some estimator variable(s) can be characterized as suboptimal.

However, claims about the deleterious effect of suboptimal estimator variables on confident suspect ID accuracy come from mock-crime studies involving very few observations, without participants being given the opportunity to opt out because they did not get a good look at the perpetrator’s face (as often happens in real-world investigations), and without being corroborated by any real-world investigations. In our view, such evidence is not yet sufficient to guide expert testimony or policy. Both lab and field data indicate that confident IDs from a pristine lineup remain highly accurate across a remarkably wide range of conditions. Whether accuracy breaks down under extreme conditions (such that $d{\prime}$ approaches 0) remains unclear because the only evidence comes from mock-crime studies that involve very few observations and that remain uncorroborated by field studies.

In terms of theory, the findings we analyzed here suggest that when encoding conditions are poor, many witnesses realize that they did not form a clear memory of the perpetrator and respond accordingly (namely, by not confidently identifying any face). These results support the likelihood ratio theory of eyewitness identification proposed by Semmler et al. (2018). This is perhaps not surprising given that likelihood ratio theories of recognition memory have been dominant in the basic science literature for decades (e.g., McClelland & Chappell, 1998; Shiffrin & Steyvers, 1997). Still, it is interesting―and is of applied relevance―that the same theory seems to apply to eyewitnesses tested using a pristine lineup.

As a reminder, everything we have presented here has to do with the initial test of a witness’s uncontaminated memory using a pristine lineup procedure. Obviously, these are not conditions that apply to every case—or even to most cases—in the real world. When a non-pristine procedure is used, such as a highly suggestive showup (an identification procedure only including the police suspect), the confidence-accuracy relationship remains strong (e.g., Eisen et al., 2017, 2022), but high-confidence accuracy (~ 80% correct) is notably lower than what is often observed with pristine lineups (~ 95% correct). In addition, it is not clear whether high-confidence accuracy for a showup is as resistant to poor estimator variable conditions as pristine lineups are. However, before considering suspect ID accuracy when memory is tested using a non-pristine procedure, it is important to first be clear about the story that applies to the pristine case.

Even when a pristine procedure is used, confident identifications can be unreliable information under certain conditions. For example, the information is unlikely to be reliable if the witness’s memory is already contaminated prior to the first test or if the suspect is included in a lineup because of his or her resemblance to a publicized photo (Smalarz, 2021). However, new model-based analyses presented here weigh against suggestions that suspect biasing factors like these may have compromised the HPD field study (e.g., Fitzgerald et al., 2023).

Finally, it is important to emphasize that every criminal case in the real world is unique and should not be mindlessly analyzed in terms of the findings we report here. For example, despite the impressive information value of confidence evident in the HPD field study and in the Ayala et al. (2025) lab study, it would be incorrect to interpret our message as “confidence trumps all,” as if no other considerations apply (Berkowitz et al., 2022; Wixted et al., 2022). Instead, each case must be analyzed on its own terms, and a considerable body of work has identified other variables that can help to discriminate accurate from inaccurate suspect identifications even when a pristine lineup is used, such as decision time and the language used by the eyewitness when expressing confidence (Ayala et al., 2025; Dodson & Dobolyi, 2016; Grabman et al., 2019; Kelso et al., 2025; Seale-Carlisle et al., 2019). Thus, while confidence serves as a valuable diagnostic tool in eyewitness identification cases, it should be considered alongside other empirically validated factors as part of a comprehensive, case-by-case evaluation of identification evidence.

## Data Availability

The data we analyzed from Ayala et al. (2025) were previously published and were made available on OSF by them here: https://osf.io/x2ru4/. The HPD field data are presented here in Table 1.

## Footnotes

1 Ayala et al. posted their data on OSF: https://osf.io/x2ru4/.

2 The insight that a signal detection model can estimate the base rate of TP lineups, as described in Wixted et al. (2016), is attributable to Professor John Dunn (University of Western Australia).

3 (Concerns have sometimes been raised about dividing TA IDs by lineup size to estimate the false alarm rate because it provides a misleading estimate when the lineup is unfair (e.g., Fitzgerald et al., 2023). We use it here because it is difficult to see how the lineups in the Ayala et al. study could be unfair).

4 As discussed later, these conclusions do not mean that case-specific details should be ignored. Real-world cases always present additional potentially relevant factors for judges or juries to consider (e.g., a lying witness, an inaccurate police report, a biased identification procedure, etc.).

5 Again, this conclusion does not apply to every real-world case. In any given case, the prosecution and defense may debate whether suspect bias exists, and the judge or jury will decide.

6 (In more detail: the sequential condition in Ayala et al. involved ~ 61% TP lineups, so we multiplied the TP frequency counts by a factor of 0.50/.61 and multiplied the TA frequency counts by a factor of 0.50/(1—.61) such that now the TP and TA frequency counts were equal (as if equal base rates had been used).

7 Somewhat confusingly, if the decision axis in a signal detection model is redefined from “strength of memory-match signal” to “log likelihood ratio,” then these same findings would imply that the confidence criteria remain essentially constant as *dʹ* changes. However, this is a technicality that does not affect the essence of our argument.
